# Rectal Traumatic Neuroma Mimicking a Malignant Mass

**DOI:** 10.7759/cureus.71531

**Published:** 2024-10-15

**Authors:** Mousa Mobarki, Sameeh Algassmi, Ali Baghdadi, Ahmed Safhi, Basim Mulaykid, Shaqraa Musawi

**Affiliations:** 1 Basic Medical Sciences (Pathology), Faculty of Medicine, Jazan University, Jazan, SAU; 2 Histopathology, Jazan Regional Laboratory, Jazan Health Cluster, Jazan, SAU; 3 Medical Laboratory Technology, College of Nursing and Health Sciences, Jazan University, Jazan, SAU

**Keywords:** anastomosis, gastrointestinal, neuroma, rectum, trauma

## Abstract

Traumatic neuromas rarely occur in the digestive system, but the biliary tree is the most common site for neuromas in the post-cholecystectomy or liver transplantation setting. Herein, we report an extremely rare rectal neuroma in a 48-year-old male with familial adenomatous polyposis (FAP) and a history of subtotal colectomy for colonic adenocarcinoma. The surgical resection was performed for clinical, endoscopic, and radiological suspicion of recurrent malignancy. Surprisingly, the resected lesion was confirmed to be a traumatic neuroma at the ileorectal anastomosis.

## Introduction

Traumatic neuroma is a reactive benign nonneoplastic lesion of the injured proximal nerve endings [1]. Traumatic neuromas usually affect the lower extremities, followed by the head and neck regions [2]. The pathogenesis of traumatic neuroma is linked to nerve injury secondary to trauma, surgery, or ischemia [1]. Intraabdominal gastrointestinal traumatic neuromas are rare and usually affect the biliary tract post-cholecystectomy or liver transplantation [1]. Herein, we report a rare case of traumatic neuroma at the site of an ileorectal anastomosis years after a subtotal colectomy for colonic adenocarcinoma and diffuse colonic polyposis in a patient with genetically confirmed familial adenomatous polyposis (FAP).

## Case presentation

**Figure 1:**
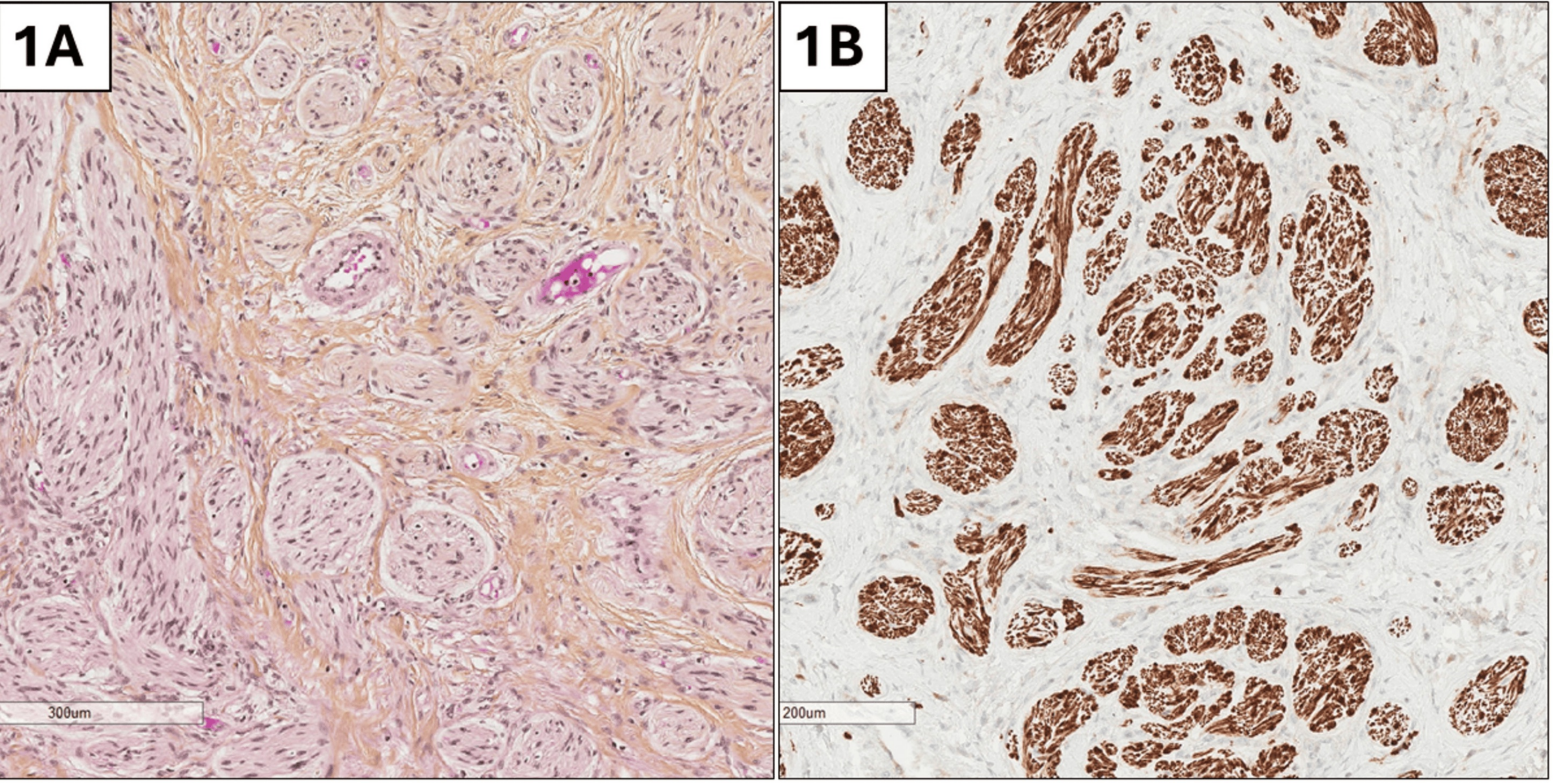
A: Microscopic image of disorganized nerve bundles in a fibroblastic collagenous stroma, consistent with traumatic neuroma; B: Positive S100 immunostaining in the traumatic neuroma lesion.

A 48-year-old male, with FAP secondary to APC gene mutation, underwent a subtotal colectomy for colonic adenocarcinoma and diffuse polyposis with ileorectal anastomosis and subsequent adjuvant chemotherapy at the age of 28 years. Then the patient was followed by regular endoscopic surveillance in which multiple duodenal adenomas with high- and low-grade dysplasia were removed along with an ampullary adenoma. No extra gastrointestinal manifestations were documented by the patient or noticed by the clinician. Routine laboratory investigations were normal. Recently, upon his regular follow-up, the patient was not complaining of anything except a nonspecific intermittent abdominal pain with no change in bowel habits, fever, or any recall of weight loss. The clinical examination was unremarkable, with a soft, non-tender abdomen. The lower endoscopic study demonstrated a suspected nongranular lesion with deep invasion at the ileorectal anastomosis along with multiple rectal polyps. The imaging studies confirmed the endoscopic suspicion with no evidence of lymph nodes or distant metastases. The treatment decision by the multidisciplinary team was a surgical removal of this suspected lesion in the setting of FAP. The patient underwent a total proctectomy with an ileostomy without any postoperative complications. Macroscopic examination of the surgical resection specimen revealed a poorly defined whitish mesenteric 2 × 1.7 cm lesion with several rectal polyps. The microscopic examination showed clusters of disorganized nerves of variable sizes in a background of dense collagenous stroma (Figure 1A). The lesion was positive for S100 immunostaining (Figure 1B), confirming the diagnosis of traumatic neuroma at the ileorectal anastomosis. No microscopic evidence of invasive colonic adenocarcinoma was detected. The patient's last follow was uneventful.

## Discussion

A traumatic neuroma, characterized by a disorganized cluster of nerve bundles formed in a reactive benign manner arising from the axons and Schwann cells, can form after trauma, surgery, or ischemia [1]. This lesion can occur anywhere, but most commonly occurs in the lower extremities and head and neck regions [2]. Traumatic neuromas are rare in the gastrointestinal tract and usually develop in the biliary system following cholecystectomy and liver transplantation [1]. Clinically, most gastrointestinal traumatic neuromas are asymptomatic. However, when gastrointestinal traumatic neuromas are symptomatic, the clinical presentation depends on the involved site, such as acute cholangitis in post-cholecystectomy traumatic neuroma involving the cystic duct or even liver transplant failure following liver transplantation [3,4]. Other sites in the gastrointestinal tract have been scarcely documented in the literature following surgical intervention, including the stomach and rectum [5-7]. Rectal traumatic neuromas are the least reported among gastrointestinal traumatic neuromas (Table 1) and are usually related to surgery [2,6,7], including our current case.

**Table 1:** Clinicopathological characteristics of reported rectal traumatic neuroma.

No	Author	Age (Years)	Gender	Primary Intervention	Time to TN diagnosis	Site	Diagnostic specimen	Histopathological features	IHC PS100
1	Jeon et al., 2016 [2]	59	Male	Ultralow anterior resection for rectal cancer	32 months	Stump of inferior mesenteric artery	Surgical biopsy	Size: 18 mm; Microscopy: disorganized nerve bundles	N/A
2	Curran et al., 2015 [6]	53	Male	Incomplete endoscopic removal of inflammatory polyp	6 years	Rectum	Transanal endoscopic microsurgery resection	Size: 10 mm; Microscopy: disorganized nerve bundles	+
3	Estifan et al., 2019 [7]	50	Female	Cap-assisted endoscopic mucosal resection	No history of previous surgery	Rectum	Cap-assisted endoscopic mucosal resection	Size: 4 mm; Microscopy: disorganized nerve bundles	+
4	Mousa et al. [current case]	48	Male	Total proctectomy	20 years	Ileorectal anastomosis	Surgical resection	Size: 20 mm; Microscopy: disorganized nerve bundles	+

The pathogenesis of traumatic neuroma is attributed to disruption of nerve continuity, resulting in reactive hyperplastic nonneoplastic proliferation arising from nerve axons and Schwan cells [1]. Several risk factors are linked to the development of traumatic neuromas, including surgical procedures, immunosuppressants, infections, foreign bodies, trauma, ischemia, and scarring [1]. Interestingly, this is in line with our current case, which developed after a subtotal colectomy.

Traumatic neuroma can be diagnosed by clinical, laboratory, and radiological correlation. However, tissue specimens are sometimes needed for diagnostic confirmation of lesions suspected of a more aggressive malignant condition [1]. In our case, the endoscopic and radiological impression suggested a recurrence of colonic adenocarcinoma in a patient with FAP; therefore, the lesion was surgically resected. Microscopically, traumatic neuromas are primarily composed of clusters of disorganized hyperplastic nerve bundles in fibroblastic collagenous stroma [1]. The lesion in our case was consistently positive for anti-PS100 on immunohistochemistry. The histopathological differential diagnosis includes neurofibromas, schwannoma, and mucosal neuromas. Neurofibromas are microscopically distinguished by the randomly mixed proliferation of Schwann cells with a few axons and mast cells with shredded collagen fibers in a myxoid stroma that can develop in the setting of neurofibromatosis type 1 [8]. The schwannoma is characterized histologically by alternating hyper- and hypocellular areas of spindle cells with Verocay bodies and is rarely associated with neurofibromatosis type 2 [8]. Mucosal neuromas are typically associated with multiple endocrine neoplasia type 2 (MEN2) and unrelated to trauma [9].

The treatment of traumatic neuroma depends mainly on the clinical presentation and includes follow-up with conservative management for clinically asymptomatic patients or surgical or interventional procedures for obstructive symptoms or to rule out any suspicion of malignancy [1], as in our case. Future studies or larger case series are necessary to gain a more comprehensive understanding of the occurrence and implications of rectal traumatic neuroma that could mimic malignancy, particularly in gastrointestinal settings and in patients with a history of gastrointestinal surgical trauma.

## Conclusions

In summary, traumatic neuroma is a non-cancerous reactive growth that may cause concern for malignancy in some situations, requiring surgical removal for diagnostic, therapeutic, and prognostic reasons. The report's purpose is to notify pathologists and clinicians about this diagnosis in the appropriate clinical context in order to prevent unnecessary treatment or intervention.
